# Diabetic Retinopathy Screening and Monitoring of Early Stage Disease in Australian General Practice: Tackling Preventable Blindness within a Chronic Care Model

**DOI:** 10.1155/2016/8405395

**Published:** 2015-12-20

**Authors:** Lisa Crossland, Deborah Askew, Robert Ware, Peter Cranstoun, Paul Mitchell, Andrew Bryett, Claire Jackson

**Affiliations:** ^1^Discipline of General Practice, School of Medicine, The University of Queensland, Level 8, Health Sciences Building, Royal Brisbane and Women's Hospital, Herston, QLD 4006, Australia; ^2^School of Public Health, The University of Queensland, Level 2, Public Health Building, Herston Road, Herston, QLD 4006, Australia; ^3^Strathpine Specialist Centre, Dixon Street, Westfield Strathpine, Strathpine, Brisbane, QLD 4500, Australia; ^4^Centre for Vision Research, Westmead Millennium Institute and Discipline of Ophthalmology and Eye Health, University of Sydney, Sydney, NSW 2006, Australia; ^5^Healthcare Improvement Unit, Healthcare Innovation and Research Branch Clinical Excellence Division, Department of Health, Queensland Government, Level 2, 15 Butterfield Street, Herston, QLD 4006, Australia

## Abstract

*Introduction*. Diabetic retinopathy (DR) is the leading cause of preventable blindness in Australia. Up to 50% of people with proliferative DR who do not receive timely treatment will become legally blind within five years. Innovative and accessible screening, involving a variety of primary care providers, will become increasingly important if patients with diabetes are to receive optimal eye care. *Method*. An open controlled trial design was used. Five intervention practices in urban, regional, and rural Australia partnered with ophthalmologists via telehealth undertook DR screening and monitoring of type 2 diabetes patients and were compared with control practices undertaking usual care 2011–2014. *Results*. Recorded screening rates were 100% across intervention practices, compared with 22–53% in control practices. 31/577 (5%) of patients in the control practices were diagnosed with mild-moderate DR, of whom 9 (29%) had appropriate follow-up recorded. This was compared with 39/447 (9%) of patients in the intervention group, of whom 37 (95%) had appropriate follow-up recorded. *Discussion and Conclusion*. General practice-based DR screening via Annual Cycle of Care arrangements is effective across differing practice locations. It offers improved recording of screening outcomes for Australians with type 2 diabetes and better follow-up of those with screen abnormalities.

## 1. Introduction

Diabetes is one of the leading causes of morbidity and mortality in the developed world. In 2013, there were an estimated 382 million diabetics globally, with the worldwide prevalence of diabetes predicted to increase to 592 million people by 2035 [1]. Of these, approximately 1 million Australian adults have been diagnosed with diabetes, including 848,000 with type 2 diabetes [2]. Diabetic retinopathy (DR) is the leading cause of preventable blindness in working age populations [3]. It is estimated that up to 50% of people with proliferative DR who do not receive timely treatment will become legally blind within 5 years [4] and although up to 98% of visual loss due to DR can be prevented with early detection and treatment, once it has progressed, vision loss is often permanent [5, 6].

The Australian healthcare system is a complex mix of public and private healthcare. The Australian Government's funding contributions include a universal public health insurance scheme, Medicare. Medicare was introduced in 1984 to provide free or subsidised treatment for care provided by health professionals such as general practitioners (GPs) and specialists [7]. The Australian National Health and Medical Research Council (NHMRC) DR guidelines provide criteria for optimal DR screening intervals [8] but less than half the Australians with diabetes receive appropriate screening, although most are receiving regular GP review [9]. The majority of current DR screening in Australia is delivered by optometrists and ophthalmologists in both private and public settings [10].

Previous research has demonstrated that general practice-based DR screening can meet NHMRC sensitivity and specificity requirements and be acceptable to both general practices and their patients with diabetes [11, 12]. This paper describes the impact of a broader research partnership between the University Queensland Discipline of General Practice, Queensland Health, the Royal Australian, and New Zealand College of Ophthalmologists (RANZCO) and Optimed camera suppliers to extend the pilot work across the state. The study aimed to trial the impact of general practice-based DR screening across a range of geographic contexts, integrated into the practices' Diabetes Annual Cycle of Care, and compared this with conventional methods of DR screening. The secondary aim was to investigate the efficacy of routine monitoring by GPs of mild to moderate DR levels with distant ophthalmic support, compared with conventional methods of DR management.

## 2. Methods

This open controlled trial was conducted in general practices throughout Queensland between February 2011 and February 2014 to compare general practice-based DR screening and monitoring with usual care over a 3-year time period. The full study protocol has been described previously [13]. The study was approved by the University of Queensland Behavioural and Social Science Ethical Review Committee.

### 2.1. Annual Cycle of Care Arrangements

Presently, all accredited Australian general practices are eligible for participation in the Diabetes Incentives Program, an initiative to support and incentivise practice activities that encourage quality care and improve access and health outcomes for patients. The Diabetes Annual Cycle of Care is Medicare-refundable for activities related to early detection of micro- and macro-vascular diabetes complications. However, ocular assessment is unclear, with no specific mention of retinopathy screening [14, 15].

### 2.2. General Practice Recruitment

The study involved 10 general practices (five in each arm), representing urban, regional, and rural areas across Queensland, with at least 50 patients with type 2 diabetes receiving regular diabetes care via Medical Benefits Schedule (MBS) supported Annual Cycle of Care arrangements. All practices had established diabetes databases. General practices were purposefully targeted to participate in this project. Intervention practices were identified first. Recruitment focused on rural towns, regional centres, and areas where populations may experience the greatest barriers to accessing appropriate DR screening. Participating general practices were required to have established recall and reminder systems for the practice Diabetes Annual Cycle of Care visits. Additional considerations for general practice recruitment included the practice having the physical space to accommodate the camera and at least one GP willing to undertake the upskilling and accreditation assessment, “read” the retinal photographs, and act as the screening “champion” within the practice. The local Division of General Practice independently matched control practices to the interventions practices by geographical region, hospital referral pathways, and size and characteristics of patients with diabetes. These practices were then invited to participate by the study team.

### 2.3. Patient Recruitment

Patients with a confirmed diagnosis of type 2 diabetes in their medical records were opportunistically recruited when they attended an intervention practice for their Diabetes Annual Cycle of Care visits, although other site specific patient recruitment processes were also used. The study was explained to patients and informed consent obtained before any data was collected or retina was photographed. Inclusion criteria were patients 18 years of age or older, with type 2 diabetes and with sufficient cognition to provide informed consent. Patients were excluded from participation if they had no perception of light in either eye, had previously diagnosed visual impairments that impacted on screening (such as cataracts), were terminally ill or deemed too unwell to participate, had a physical or mental disability that prevented either screening or treatment, or were already under the care of an ophthalmologist for treatment and follow-up of DR.

### 2.4. Diabetic Retinopathy Screening Pathways

**Figure 1:**
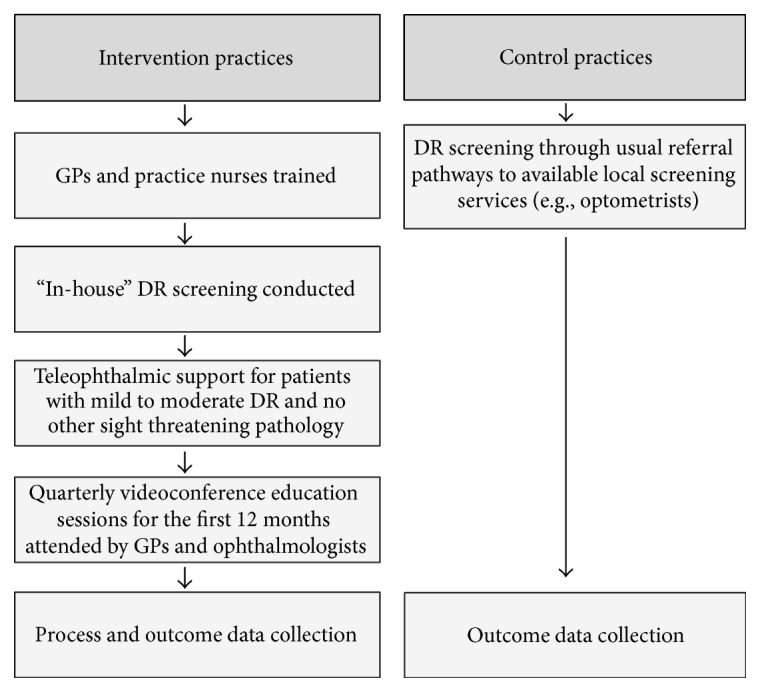
Study processes for intervention and control practices.

Figure 1 provides a graphical representation of the study design. Each intervention practice received a non-mydriatic camera fully installed, with staff training on its use and maintenance. The participating GPs in the intervention practices completed a four-hour on-line DR upskilling program through the University of Queensland Masters of Medicine (General Practice) Program, followed by an accreditation assessment through RANZCO Queensland Faculty [13]. All those patients confirmed through their clinical notes as diagnosed with type 2 diabetes and attending intervention practices for Diabetes Annual Cycle of Care assessments were offered “in-house” DR screening. Each intervention practice was partnered with a distant ophthalmologist for the duration of the study. Patients without DR were rescreened at a later date according to NHMRC Guidelines [8]. Patients with mild-moderate DR, diagnosed by the screening GP, were reviewed with the practice partner ophthalmologist through regular teleconferences and/or e-contact. Ongoing management regarding referral or later reassessment was agreed and recorded. Patients with severe DR, other pathologies, or conditions precluding quality imaging were referred immediately to an ophthalmologist.

DR screening in the five control practices was undertaken as part of the Annual Cycle of Care via usual referral pathways. In the control practices “usual referral pathways” involved a referral (via either reminder letter or verbal reminder to the patient) that screening was due. Patients were advised to attend their usual screening service.

### 2.5. Sample Size Calculation

Based on practice self-reports, it was calculated that 40% of control practice participants would receive timely and appropriate DR screening. A conservative assumption was made of an intracluster correlation of 0.25 and that the median number of participants enrolled at each practice would be 100. Therefore, 10 participating practices would have 80% power to detect a between-group difference of 42% or greater (i.e., an appropriate screening rate of 82% or greater in participants attending an intervention practice).

### 2.6. Outcome Assessment

The main outcomes measures reported in this paper are the percentage of patients with type 2 diabetes in the intervention practices who received timely and appropriate DR screening, compared to rates in the control practices, and the proportion of patients with identified mild-moderate level DR who attended for review appointments in the intervention practices, compared to rates in the control practices.

### 2.7. Statistical Analysis

Summary statistics are presented as mean (standard deviation) for continuous variables and frequency (percentage) for categorical variables. The association between practice (control/intervention) and outcome was investigated using the chi-square test. Analyses were completed using Stata statistical software v.12.0 [16].

## 3. Results

Ten general practices, five intervention and five control, from across Queensland, participated in the trial. Practices were classified using the Rural Remote and Metropolitan Area (RRMA) index [17] and represented metropolitan, large rural and other rural locations. The RRMA index is based on distance to service centres as well as distance from other sites. It is still a preferred classification index used in health or community-related research and was thus used in this study. Practice characteristics were similar to those described nationally, based on comparisons with recent reports from the Australian Institute of Health and Welfare (AIHW) and Bettering the Evaluation and Care of Health (BEACH) [18, 19].

There were a total of 447 eligible patients recruited in the intervention practices and 577 patients recruited in the control practices via a deidentified chart audit. Control practice patients had a mean age of 66.1 years (standard deviation of 12.8) while comparative figures from the intervention practice patients were 68.3 (12.7) years. There were comparable numbers of male and female patients in the intervention (51% male) and control (55% male) practices. These figures are representative of the Australian population with type 2 diabetes for both age and gender [19]. Descriptions of intervention and control patients were also completed in relation to the three guideline variables most strongly linked with DR progression, namely, duration of disease, hypertension, and HbA1c [9, 20, 21]. Duration of disease was calculated based on the documented date of diagnosis. Blood pressure and HbA1c values were collected from the clinical notes (details in Appendix 1 in the Supplementary Material available online at http://dx.doi.org/10.1155/2016/8405395). For patients with more than one screen for the DR study, only the most recent screening results were used.  Table 1 provides a summary of the key characteristics of the eligible patients from intervention and control practices (further details are provided in Appendix 1). The differences between the ineligibility numbers were largely to do with the higher number of patients with other complicating conditions. These patients were (i) already diagnosed with DR and under the care of a specialist; (ii) already under the care of a specialist and/or hospitalised for other conditions; (iii) had diagnosed visual or physical impairments that immediately precluded them from screening. There were also a high number of transient patients (e.g., those passing through areas during extended vacation and travel periods and also temporary residents). This was particularly so in the rural and regional areas.

NHMRC-appropriate screening rates were 100% across eligible patients in intervention practices, compared with 22–53% in control practices (Table 2). This included two key subgroups of patients, namely, those who were referred directly to an ophthalmologist as it was deemed unlikely that a reasonable image could be taken due to complicating physical factors, and those who had significant other pathology detected and also were referred directly to an ophthalmologist for care. Only one intervention practice patient had a referral for screening noted but no screening outcome recorded.

All intervention practices achieved screening rates that were greater than the national population average of 48% [9]. These practices were also significantly more likely to have records of screening referral or reminder than their matched controls (*P* < 0.01). These included patients with physical issues which did not fully prevent screening but made it difficult to get an adequate image for review and it was determined that a readable image was unlikely and also patients with significant other pathology. Both these subgroups of patients were referred directly to an ophthalmologist and their subsequent screening outcome was recorded.

A total of 3/447 (0.6%) of patients in the intervention practices and 2/577 (0.3%) in the control practices had screening outcomes recording severe DR. In the intervention practices these patients were referred directly to an ophthalmologist for ongoing care. For the subset of patients with documented mild-moderate DR, four of the five intervention practices had recorded appropriate follow-up management for 100% of patients (Table 3). For three control practices, appropriate follow-up ranged from 27 to 57% of patients with mild-moderate DR. In the remaining two control practices there was no recorded timely follow-up.

Overall, there were 39/447 (8.7%) of patients diagnosed with mild-moderate DR in the intervention practices, of whom 37/39 (95%) had appropriate follow-up recorded. This was compared with 31/577 (5%) patients diagnosed with mild-moderate DR in the control practices, of whom 9/31 (29%) had appropriate follow-up recorded. During the review of the control practice diabetes registers, it was noted that the average time of follow-up recorded was 3 years. One intervention practice (classified as a large rural centre) demonstrated a lower rate of appropriate follow-up for patients diagnosed with mild-moderate DR (71%) compared with other intervention practices which all achieved 100%.

## 4. Discussion

Patients in intervention practices were more likely to have a screening referral or reminder recorded and those diagnosed with DR were more likely to have evidence of timely and appropriate follow-up, when compared with control practices. Mean patient DR screening rates in control practices were similar to nationally reported rates [9], whilst those in intervention practices were significantly higher, reaching 100% of eligible patients in all five practices. This demonstrates the large improvement possible in NHMRC-appropriate DR screening, when DR screening is incorporated proactively into regular practice chronic disease initiatives such as the preexisting GP Diabetes Annual Cycle of Care assessment. Whilst all facets of the intervention (i.e., practice staff training, teleophthalmic support, and GP education) were integral to the high screening rates achieved in the intervention practices, results also underline the benefits of* empanelment* in chronic disease management, a process of assigning patients to individual primary care teams with sensitivity to patient and family preference. Empanelment has been seen as the key to continuity of care, creating a focus on a population of patients to ensure that every established individual receives optimal care, whether he/she regularly comes in for visits or not [22].

The approach taken within this study also inadvertently led to a focused review and update of diabetes registers in the intervention practices. Intervention practice registers more accurately reflected the proportion of patients with type 2 diabetes that required DR screening and, following this, undertook proactive invitations for screening and rigorous patient follow-up. This resulted in the accurate capture of patients targeted for screening in all five practices. As a* population-based* rather than individual patient-based approach, it also allowed both a concentrated practice-wide focus on DR screening and an exact assessment of the screening denominator, which more accurately excluded patients already screened or those who were ineligible for screening. Control practices, without such measures, identified the practice's recorded screening referral rate to be far greater than that of recorded outcome. As screeners external to the practice rarely returned a record of screening outcome, patients may not have attended for screening, or the practice may have had an inaccurate screening denominator. Control practices and their patients were reliant on third parties to coordinate ongoing screening and review and there was no central repository of outcome and management intent. The single control practice with a demonstrably higher recorded DR screening rate (57%) had a close working relationship with their local optometrists, underlining the importance of a formal recorded linkage between screening result and ongoing management and referral and the benefits of close working relationships between general practice and optometry.

Overall, results demonstrate the powerful impact of incorporating DR screening into the process for all other annual diabetes micro- and macro-vascular review. Whilst practice training, process review, and infrastructure adjustment required initial attention, DR screening fitted neatly into established processes for both patients and practice. The link between practice screeners and their “buddy” ophthalmologist was an important part of this. It allowed rapid access to advice, education, and follow-up support for patients with abnormal images. It also resulted in 100% of follow-up management and outcome recorded in the patient GP file in four out of five intervention practices, a critical element in ongoing management and review. Whilst an online secure videoconferencing program was initially used for training and establishment of the “buddy” ophthalmology network, GP screeners and ophthalmologists universally moved to email communication due to its ease, quality of image transfer, and opportunity for “non-real-time” access and response times.

These study findings may inform Australian state and federal health department planning to better enable DR screening in primary care. The model supports greater collaboration between primary care and specialist providers, contributes to the development of sustainable models of care, and informs design and deployment of fit-for-purpose technologies to capture, share, and report on retinal images. Queensland Health is currently exploring opportunities to enhance its asynchronous telehealth capability to meet this clinical need. The issues related to the impacts of embedding DR screening in practice workflow, the impact of individual elements of the intervention (education, training, and support), and the role and impact of the GP screening “champions” were investigated in the study through the collection of qualitative data. These results have been published in detail elsewhere. The findings presented here also reinforce the importance of the patient's “medical home” in ongoing chronic disease management [23]. An established process for patient identification, recall, annual review, and intervention, where necessary, is critical to optimal long-term management and minimisation of morbidity. Fragmented “ad hoc” arrangements are expensive, often duplicative, and frequently miss patients most at-risk. The study demonstrates the impact of single point accountability for both screening and ongoing management of abnormality, with highly significant differences between intervention and control practices in recorded follow-up for patients with established DR. The Consultation paper for the development of the Australian National Diabetes Strategy, released in April 2015, calls for programs, monitoring and reporting across primary health networks and the health system more generally encompassing eye damage and blindness [24]. General practice may be an important investment in this regard.

An assessment of the economic elements of the model has been completed and submitted for publication elsewhere. The Medical Services Advisory Committee (MSAC) has recently recommended specific Medicare funding for community DR screening. In Australia, this would reimburse the main screening elements currently not covered, namely, GP review time, and also contribute toward the costs of the non-mydriatic cameras.

*Limitations of the Study*. This work adds to the previous studies confirming the accuracy of DR screening within general practice and applies it to Queensland general practices of differing sizes, located across a range of RRMA classifications, over a three-year observation period. However, although representative, the study involved only a small number of Queensland practices. It also utilised DR screening results recorded in the patients' general practice records to calculate screening frequency. Thus, some patients may potentially have had screening, but its occurrence was unknown and thus unable to be captured and included in ongoing chronic disease management.

There was also an overall poor quality of the diabetes registers. These contained incomplete information, inactive patients, and prediabetic and gestational diabetes mellitus patients that made the identification of relevant information challenging. In addition, there were a number of patients who did not meet the eligibility criteria in the intervention practices. These included transient patients in the rural areas as well as those who were diagnosed with other illnesses or conditions and placed in hospital or under another specialist care. Intervention practices have continued DR screening since study conclusion, demonstrating the acceptability of the intervention and potential to embed research into practice.

## 5. Conclusion

Australia has suboptimal national screening rates for DR, a common cause of preventable blindness. This study demonstrates the inclusion of DR screening via non-mydriatic retinal photography into the annual GP Diabetes Cycle of Care to be highly effective across differing practice size and location. General practice-based DR screening via Annual Cycle of Care arrangements offers both improved recording of screening outcomes for Australians with type 2 diabetes and better follow-up especially of those with screen abnormalities, due to the close links between ophthalmologists and GPs. Better utilisation of existing community chronic disease management infrastructure, incorporation of DR screening into already operational diabetes care regimens, and appropriate incentives could significantly reduce the burden of preventable blindness in rural, regional, and urban Australia.

## Supplementary Material

Appendix 1 provides additional information on the eligible patients in both intervention and control practices including comparisons of NHMRC guideline variables most strongly linked with DR progression and also most routinely recorded by the practices; namely duration of disease, HbA1c and hypertension.

## Figures and Tables

**Table 1:** Characteristics of intervention and control practice populations.

	Patients *N*	Male *n* (%)	Female *n* (%)	Mean age in years (±SD)^†^	Mean HbA1c (±SD)^†^	Mean SBP^*∗*^ (±SD)^†^	Mean DBP^‡^ (±SD)^†^	Median duration of disease in years (interquartile range)^#^
Intervention	447	228 (51)	219 (49)	68.3 (12.7)	7.4 (1.6)	132.6 (16.9)	73.9 (11.3)	6 (3, 11)
Control	577	318 (55)	259 (45)	66.1 (12.8)	7.7 (2.3)	135.3 (18.3)	77.2 (11.3)	7 (2, 11)

^*∗*^Systolic blood pressure.

^‡^Diastolic blood pressure.

^†^Age; HbA1c, SBP, and DBP reported as the mean plus or minus (±) SD (standard deviation).

^#^Duration of disease reported as median (interquartile range).

Number of observations for SBP, DBP and duration of disease was 400 (in the intervention group) and 431 (in the control group).

**Table 2:** Screening rates achieved.

RRMA scores 1 = metropolitan 2 = other metropolitan 3 = large rural centre	Total eligible		Referral or reminder		Screening outcome		
	Study population		for screening recorded		recorded		
	(*N*)		*n* (%)		*n* (%)		
4 = small rural centre 5 = other rural centre	Intervention	Control	Intervention	Control	Intervention		Control
1	174	181	174 (100)	101 (56)	173^*∗*^ (99)		40 (22)
					Image unlikely^†^	Other pathologies^‡^	
					4	2	

4	79	131	79 (100)	59 (45)	79 (100)		69 (53)
					Image unlikely	Other pathologies	
					3	0	

1	78	81	78 (100)	51 (63)	78 (100)		24 (30)
					Image unlikely	Other pathologies	
					0	0	

3	70	108	70 (100)	90 (83)	70 (100)		33 (31)
					Image unlikely	Other pathologies	
					2	0	

3	46	76	46 (100)	59 (78)	46 (100)		27 (36)
					Image unlikely	Other pathologies	
					2	1	

Total	**447**	**577**	**447 (100)**	**360 (62)**	**446 (100)**		**193 (33)**

^*∗*^1 patient had a referral noted for which there was no recorded outcome.

^†^“Image unlikely” refers to patients who had minor physical issues which influenced the ability to get readable images and so were referred directly to the ophthalmologist.

^‡^“Other pathologies” refers to patients with pathology other than DR which required direct referral to the ophthalmologist.

**Table 3:** Frequency (percentage) of monitoring mild-moderate DR achieved.

RRMA scores 1 = metropolitan 2 = other metropolitan 3 = large rural centre	Patients screened		DR		Follow-up recorded	
	(screening outcome recorded)		(mild-mod.)		≤12 months^*∗*^	
	*n* (%)		*n* (%)		*n* (%)	
4 = small rural centre 5 = other rural centre	Intervention	Control	Intervention	Control	Intervention	Control
1	173 (100)	40 (22)	9 (5)	3 (8)	9 (100)	1 (33)
4	79 (100)	69 (53)	7 (9)	15 (22)	7 (100)	4 (27)
1	78 (100)	24 (30)	8 (10)	3 (13)	8 (100)	0
3	70 (100)	33 (31)	7 (10)	7 (21)	5 (71)	4 (57)
3	46 (100)	27 (36)	8 (17)	3 (11)	8 (100)	0

^*∗*^Average time to follow-up rescreen in the control practices = 2.5–3 years as indicated in review of diabetes registers.
