# Supplementary material for: Diabetic Retinopathy Screening and Monitoring of Early Stage Disease in Australian General Practice: Tackling Preventable Blindness within a Chronic Care Model
Source: J Diabetes Res. 2015 Dec 20;2016:8405395. doi: 10.1155/2016/8405395 (PMC4698989; doi:10.1155/2016/8405395)
Supplement: Supplementary file 1 — Appendix 1 provides additional information on the eligible patients in both intervention and control practices including comparisons of NHMRC guideline variables most strongly linked with DR progression and also most routinely recorded by the practices; namely duration of disease, HbA1c and hypertension. [file 8405395.f1.pdf]

Appendix 1

Table 1 Eligible patients: Intervention and control practices

Practice RRMA<br>1= Metropolitan<br>2= Other metropolitan<br>3= Large rural centre<br>4= Small rural centre<br>5= Other rural centre	Total Diabetes patients February 1, 2011		Refused*		Ineligible		Total diabetes study patients	
	Intervention	Control	Intervention	Control	Intervention	Control	Intervention	Control
**1**	268	213	0	0	94	32	**174**	**181**
**1**	144	142	10	0	66	61	**78**	**81**
**3**	143	172	32	0	38	64	**70**	**108**
**3**	137	89	26		68	13	**46**	**76**
**4**	105	148	6	0	20	17	**79**	**131**
**TOTAL**							**447**	**577**

*Includes patients who elected to receive screening from services outside their general practice

Progression of Disease

The following three tables focus on NHMRC guideline variables most strongly linked with DR progression and also most routinely recorded by the practices; namely (i) duration of disease; (ii) HbA1c; and (iii) hypertension [1, 2, 3]. A the time of our study there were no 'standard' definitions of these variables provided in the Guideline, thus we utilised Mohammed et al's (2007) systematic review and Schiller et al's paper on key risk factors for DR to provide the most recent and comprehensive definitions of 'poorly controlled' diabetes, namely: glycaemic control as HbA1c ≥7%; blood pressure (BP) of >150/>90 mmHg and a duration of disease of ≥10 years which determined the those patents requiring annual versus biennial screening [2, 3]. Duration of disease was calculated based on the documented date of diagnosis. Most recent BP and HbA1c values were collected from the clinical notes. Additional risk variables including ethnicity, dyslipidaemia and renal function were not included as they were not routinely recorded by all practices.

Table 1 Duration of disease

	**Duration of disease** N (%)		
**Practice**	<10years	≥10years	Total
Intervention patients	249 (65)	133 (35)	382* (100)
Control patients	294 (68)	140 (32)	434± (100)
**Total**	543 (67)	273 (33)	816 (100)

Pearson chi square(1) = 0.5975

P-value = 0.440

*Duration of disease data were available for 382/447 intervention patients (no duration of disease available for 65 intervention patients).

±Duration of disease data were available for 434/577 control patients (no duration of disease available for 143 control patients).

Table 2 HbA1c

	HbA1c N (%)		
	**<7**	**≥7**	**Totals**
Intervention patients	217 (50.1)	216 (49.8)	433* (100)
Control patients	254 (45.8)	300 (54.1)	554± (100)
**Totals N (%)**	471 (47.7)	516 (52.2)	987 (100)

Pearson chi square(1) = 1.7738

P-value = 0.183

*HbA1c data were available for 433/447 intervention patients (no HbA1c recorded within study period for 4 intervention patients).

±HbA1c data were available for 554/577 control patients (no HbA1c recorded within study period for 23 control patients).

Table 3 Blood pressure: Systolic

	Systolic blood pressure N (%)		
	**≤150**	**>150**	**Totals**
Intervention patients	329 (82.4)	70 (17.5)	400* (100)
Control patients	338 (78.4)	93 (21.5)	431± (100)
**Totals N (%)**	667 (80.3)	163 (19.6)	831 (100)

Pearson chi square(1) = 2.1363

P-value = 0.144

Table 4 Blood pressure: Diastolic

	Diastolic blood pressure N (%)		
	**≤90**	**>90**	**Totals N (%)**
Intervention patients	366 (91.5)	34 (8.5)	400* (100)
Control patients	380 (88.1)	51 (11.8)	431± (100)
**Totals N (%)**	746 (89.7)	85 (10.2)	831 (100)

Pearson chi square(1) = 2.5098

P-value = 0.113

*Blood pressure (BP) data were available for 400/447 intervention patients (no BP data for 16 intervention patients).

±BP data were available for 431/577 control patients (no BP data for 146 control patients).

Appendix 1 References

1. McCarty D, Fu C, Harper CA, Taylor HR, McCarty CA. Five-year incidence of diabetic retinopathy in the Melbourne Visual Impairment Project. *Clin Experiment Ophthalmol* 2003; 31: 397-402.
2. Quresh M, Gillies MC, Tong TY. Management of Diabetic Retinopathy: A Systematic Review. *JAMA* 2007; 298(8): 902-916.
3. Schiffelers RM, Fens MH, van Blijswijk JM, Bink DI, Storm G. Targeting the retinal microcirculation to treat diabetic sight problems. *Expert Opin Ther Targets* 2007; 11 (11): 1493-1502.
